# A cluster randomized controlled trial to assess the impact on intimate partner violence of a 10-session participatory gender training curriculum delivered to women taking part in a group-based microfinance loan scheme in Tanzania (MAISHA CRT01): study protocol

**DOI:** 10.1186/s12905-018-0546-8

**Published:** 2018-04-02

**Authors:** Sheila Harvey, Shelley Lees, Gerry Mshana, Daniel Pilger, Christian Hansen, Saidi Kapiga, Charlotte Watts

**Affiliations:** 1 Department of Global Health and Development, London School of Hygiene & Tropical Medicine, 15-17 Tavistock Place, London, WC1H 9SH UK; 2 Mwanza Intervention Trials Unit, PO Box 11936, Mwanza, Tanzania; 3 National Institute for Medical Research, PO Box 1462, Mwanza, Tanzania; 4 Department of Infectious Disease Epidemiology, London School of Hygiene & Tropical Medicine, Keppel Street, London, WC1E 7HT UK

**Keywords:** Maisha, Intimate partner violence, Cluster randomized controlled trial, Qualitative, Microfinance, Gender training, Violence prevention, Tanzania, Africa

## Abstract

**Background:**

Worldwide, almost one third (30%) of women who have been in a relationship have experienced physical and/or sexual violence from an intimate partner. Given the considerable negative impacts of intimate partner violence (IPV) on women’s physical health and well-being, there is an urgent need for rigorous evidence on violence prevention interventions.

**Methods:**

The study, comprising a cluster randomized controlled trial (RCT) and in-depth qualitative study, will assess the impact on women’s past year experience of physical and/or sexual IPV of a participatory gender training curriculum (MAISHA curriculum) delivered to women participating in group-based microfinance in Tanzania. More broadly, the study aims to learn more about the factors that contribute to women’s vulnerability to violence and understand how the intervention impacts on the lives of women and their families. Sixty-six eligible microfinance loan groups are enrolled and randomly allocated to: the 10-session MAISHA curriculum, delivered over 20 weeks (*n* = 33); or, to no intervention (*n* = 33). Study participants are interviewed at baseline and at 24 months post-intervention about their: household; partner; income; health; attitudes and social norms; relationship (including experiences of different forms of violence); childhood; and community. For the qualitative study and process evaluation, focus group discussions are being conducted with study participants and MAISHA curriculum facilitators. In-depth interviews are being conducted with a purposive sample of 18 participants. The primary outcome, assessed at 24 months post-intervention, is a composite of women’s reported experience of physical and/or sexual IPV during the past 12 months. Secondary outcomes include: reported experience of physical, sexual and emotional/psychological IPV during the past 12 months, attitudes towards IPV and reported disclosure of IPV to others.

**Discussion:**

The study forms part of a wider programme of research (MAISHA) that includes: a complementary cluster RCT evaluating the impact of delivering the MAISHA curriculum to women not receiving formal group-based microfinance; an economic evaluation; and a cross-sectional survey of men to explore male risk factors associated with IPV. MAISHA will generate rigorous evidence on violence prevention interventions, as well as further insights into the different forms and consequences of violence and drivers of violence perpetration.

**Trial registration:**

ClinicalTrials.gov ID: NCT02592252, registered retrospectively on 13 August 2015.

**Electronic supplementary material:**

The online version of this article (10.1186/s12905-018-0546-8) contains supplementary material, which is available to authorized users.

## Background

Violence against women and girls is a major global public health and development concern. Empowering women and promoting gender equality is one of the 17 sustainable development goals outlined in the United Nations 2030 Agenda on Sustainable Development, which was adopted by countries in 2015. Ending all forms of discrimination against women and girls, including physical and sexual violence and other forms of abuse, is not only a human right issue but also crucial to accelerating sustainable development [1].

The past decade has seen a rapidly growing body of research on violence against women. Worldwide, almost one third (30%) of women who have been in a relationship have experienced physical and/or sexual violence by an intimate partner. The negative impacts of intimate partner violence (IPV) on women’s physical and mental health are considerable [2] and the impact on their children is of increasing concern, given that co-occurrence of exposure to IPV and other types of child maltreatment is high [3]. The World Health Organisation (WHO) has highlighted the urgent need for evidence on effective violence prevention interventions [4]. Although evidence is now starting to emerge, rigorous data on what works to prevent violence remain scarce. Data are highly skewed towards studies conducted in high-income countries with intervention research focused more on response than prevention [5].

One example of an intervention that aims to prevent women’s experience of IPV is the Intervention with Microfinance for AIDS & Gender Equity (IMAGE), which was developed in rural South Africa and combines group-based microfinance with a participatory gender and HIV training programme. In a cluster randomised controlled trial (RCT), IMAGE was shown, over a two-year period, to reduce women’s past year experience of physical and/or sexual IPV by 55% [6]. In addition, levels of household poverty were significantly reduced and participants were more empowered as evidenced by greater self-confidence, autonomy in decision making, and increased ability to challenge gender norms when compared with women in the control population [7]. These findings have led to national policy change and the formal inclusion of microfinance and the empowerment of women into the South African Government’s Strategic Plan for HIV/AIDS. Regional and international policy makers have asked whether, with appropriate national level refinement and adaptation, the IMAGE model would achieve the same level of impact if it was implemented in other sub-Saharan African settings.

High rates of IPV have been reported in Tanzania – the WHO multi-country study on women’s health and domestic violence found that almost 30% of ever-partnered women in a rural area of Tanzania had experienced physical and/or sexual violence from a partner in the year prior to the survey [8]. Ahead of setting up the MAISHA study to replicate the IMAGE study in Tanzania, a participatory social mapping study (unpublished) was conducted, comprising participatory group discussions and transect walks in a sample of neighborhoods in Mwanza city, northwestern Tanzania. The objectives of the study were to determine: 1) social and economic boundaries and activities in the study communities; 2) types and functioning of microfinance entities in the study communities; and, 3) feasibility of recruiting the required numbers of study participants and retaining them for over a year. In all the neighborhoods studied, both informal and formal microfinance activities were reported. Informal microfinance is initiated by neighbors (i.e. people who know each other) and involves small loans with no formal membership or loan records. Formal microfinance, delivered by developmental non-governmental organisations, requires registration with the organisation and involves relatively large loans with fixed interest rates. The social mapping study indicated that formal microfinance is not usually delivered to the poorest of the poor and that most women who take formal microfinance loans tend to come from households that are able to meet their basic daily needs and may even have accumulated some assets. It seems therefore, that women who do and do not engage in formal microfinance activities are probably different populations. Given this, the MAISHA study comprise two cluster RCTs to evaluate the impact of a participatory gender training curriculum on women’s past year experience of IPV. The first RCT (MAISHA CRT01), described in this paper, seeks to evaluate the impact of the curriculum delivered to women in established formal microfinance loan groups in Tanzania. The research question being addressed is: do women in established formal microfinance loan groups, who participate in a participatory gender training curriculum, experience lower levels of past year IPV compared with women in established formal microfinance loan groups who do not? The study is being conducted in collaboration with the Bangladesh Rural Advancement Committee (BRAC), which is one of the leading microfinance providers in Mwanza and across Tanzania. The second RCT (MAISHA CRT02), described in a separate paper, seeks to evaluate the impact of the same curriculum delivered to women in newly-formed groups who are not engaged in formal group-based microfinance.

MAISHA is being implemented by the Tanzanian National Institute for Medical Research (NIMR), Mwanza Intervention Trials Unit (MITU) and London School of Hygiene & Tropical Medicine (LSHTM).

### Aim and objectives

The overall aim of the study is to assess the impact on IPV of a participatory gender training curriculum (the MAISHA curriculum) delivered to women taking part in a formal group-based microfinance scheme. The primary objective is to assess the impact on women’s experience of physical and/or sexual IPV during the past 12 months. The secondary objectives are to assess the impact on:

- different forms of IPV – physical, sexual and emotional/psychological;
- women’s attitudes towards the acceptability of IPV; and
- women’s disclosure of violence to others.

The study also seeks, through an in-depth qualitative study, to:

- learn more about the factors that contribute to women’s vulnerability to violence; and
- to understand how the intervention impacts on the lives of participants and their families.

**Fig. 1:**
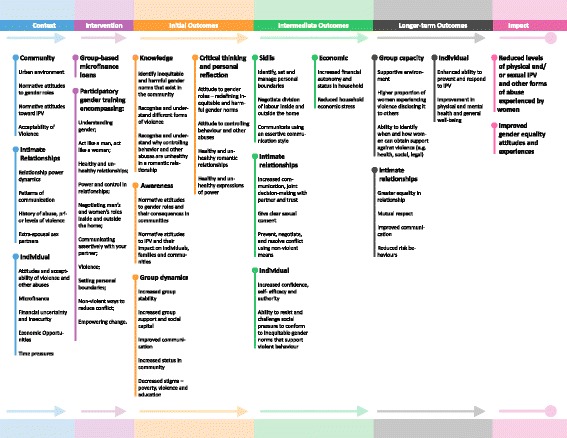
Theory of change model

The theory of change model (Fig. 1) maps out the key contextual factors that may influence the impact of the intervention, the components of the intervention, the expected initial, intermediate and longer-term outcomes of the intervention and the overall impact the intervention is designed to have on women in Tanzania.

## Methods/design

### Study design and setting

This is a mixed methods study comprising a cluster RCT with a complementary in-depth qualitative study and an integrated process evaluation. The study is being conducted in Mwanza city, in northwestern Tanzania. In collaboration with BRAC, established microfinance loan groups in Mwanza city are being identified and assessed for eligibility to take part. Each member of a microfinance loan group is required to pay a deposit before receiving their first loan. They are also required to contribute a small payment each week as a social security deposit. The interest rate is fixed at 25%. The group meets every week to repay part of the loan with a maximum loan repayment time of six months. If an individual member of the group is unable to contribute her share of the loan repayment, the other members of the group must cover this.

### Eligibility criteria

Established microfinance loan groups that meet the following criteria are eligible for inclusion in the study:

1. there are less than 30 active members in the group;
2. there is a good attendance (repayment) record based on BRAC records; and
3. a minimum of 70% of active members consent to take part in the study, that is they:
  - demonstrate comprehension of the study procedures;
  - are willing to undergo the study procedures, including attending all 10 sessions of the MAISHA curriculum, if randomly assigned to this arm of the study; and
  - have signed an informed consent form.

For each microfinance loan group enrolled, only women within the group who consent to take part, undergo study procedures.

### Intervention and comparator

The group-based microfinance loans are delivered by BRAC with no involvement from the MAISHA study team. Microfinance groups allocated to the control arm continue to meet every week for loan repayments following BRAC procedures. Although the MAISHA team continues to keep in regular contact with the groups (to minimize losses to follow-up), there is no further intervention. Microfinance groups allocated to the intervention arm also continue to meet every week for loan repayments. In addition, on alternate weeks, either before or after the loan group meeting, they receive the MAISHA curriculum – *Wanawake na Maisha* (which means “women and life” in Swahili). The curriculum comprises 10 sessions and was developed for the MAISHA study, by EngenderHealth (an international non-profit organisation focussing on family planning, maternal health, HIV and AIDS and gender equity) in collaboration with LSHTM and MITU. Some of the curriculum activities for *Wanawake na Maisha* were adapted from other curricula [6, 9–13], including the *Sisters for Life* curriculum developed for IMAGE in South Africa [6]. The overall aim of the MAISHA curriculum is that, after completing the 10 sessions, participants will have developed skills to help them minimize, and potentially prevent, IPV within intimate relationships, as well as having increased capacity to defend themselves against IPV and the negative consequences resulting from IPV. The specific objectives of the curriculum are detailed in Table 1.

**Table 1:** Objectives of The MAISHA curriculum (*Wanawake Na Maisha*)

Objective number	Intended outcome for participants is that they should be able to:
1	Identify inequitable and harmful gender norms that exist in their community, especially those norms that contribute to IPV
2	Explain how abiding to inequitable and harmful gender norms has health and social costs to women, men, families and the community
3	Re-define inequitable and harmful gender norms into equitable and healthy alternatives
4	Describe the characteristics of healthy and unhealthy romantic relationships
5	Explain why controlling and abusive behaviour is unhealthy in a romantic relationship
6	Explain healthy and unhealthy expressions of power
7	Identify, set and manage personal boundaries
8	Negotiate division of labour inside and outside the home
9	Communicate using an assertive communication style
10	Identify different forms of violence including emotional, physical, economic and sexual
11	Explain the impact of intimate partner violence on the health and wellbeing of women, men, families and communities
12	Give clear sexual consent
13	Prevent, negotiate and resolve conflict using non-violent means
14	Resist and challenge social pressure to conform to inequitable gender norms that support violent behaviour
15	Identify when and how women can obtain support against violence (e.g. health, social, legal, etc.), if needed

The MAISHA curriculum is delivered over 20 weeks. Each of the 10 sessions (outlined in Fig. 1) is approximately an hour and a half to two hours giving a total time of approximately 20 h. Each session is participatory and comprises: giving information to participants, small group activities and group discussions, and ending with a take home assignment designed to encourage participants to practice the skills covered during the session. The MAISHA curriculum is delivered by trained facilitators following the MAISHA curriculum manual, which provides detailed guidance for each session. The manual includes tips and notes for the facilitators, including examples of group ice-breakers and energisers. The facilitators have been trained by EngenderHealth to facilitate the MAISHA curriculum which included: gender equitable behavior and attitudes; managing group dynamics (including emotional reactions and disclosure of sensitive information); establishing a safe and comfortable learning environment; and encouraging all participants to take part in discussions. In addition, the training also included discussions around beliefs, including: the belief that intimate relationships should never be coercive, exploitative or abusive; belief in the importance of gender equity and women’s rights; and belief that inequitable gender norms can be changed.

Ongoing training of the MAISHA curriculum facilitators, including practicing facilitation skills through role play, is supported by MITU and LSHTM. The MAISHA curriculum facilitators are not involved in collection of baseline data or any outcome assessments for the study.

### Outcomes

The primary outcome is a composite of women’s reported experience of physical and/or sexual IPV during the past 12 months and is assessed via a face-to-face interview at 24 months post-intervention (29 months post-randomization). The secondary outcomes, also assessed at 24 months post intervention, are women’s reported experience of specific forms of IPV during the past 12 months, as follows:

- physical IPV;
- sexual IPV; and
- emotional/psychological abuse.

Other secondary outcomes are:

- women’s attitudes towards the acceptability of IPV; and
- women’s disclosure of violence to others – for those who report physical and/or sexual IPV during the past 12 months.

Table 2 details the questions asked to assess the different forms of IPV, which have been adapted from the WHO Violence Against Women instrument [8].

**Table 2:** Questions used to assess different forms of intimate partner violence experienced by women taking part in the MAISHA study (taken from the WHO Violence Against Women instrument [8])

Type of violence	Questions
Physical violence	Has your current partner or any other partner ever:
	1. Slapped you or thrown something at you that could hurt you?
	2. Pushed you or shoved you or pulled your hair?
	3. Hit you with his fist or with something else that could hurt you?
	4. Kicked you, dragged you or beaten you up?
	5. Choked or burnt you on purpose?
	6. Threatened to use or actually used a gun, knife or other weapon against you?
Sexual violence	Have you ever had sexual intercourse with your current partner or any other partner:
	1. After he forced you by threatening you, holding you down or hurting you in some way?
	2. When you did not want to because you were afraid that your partner would hurt you or someone you cared about if you refused?
	3. When you did not want to because you were afraid that your partner would leave you or take another girlfriend if you refused?
Controlling behavior	Thinking about your (current or most recent/past) partner, would you say it is generally true that he:
	1. Tries to keep you from seeing your friends?
	2. Tries to restrict contact with your family of birth?
	3. Insists on knowing where you are at all times?
	4. Is jealous and gets angry if you speak with another man?
	5. Is often suspicious that you are unfaithful?
Economic abuse	Thinking about your (current or most recent/past) partner, would you say it is generally true that he:
	1. Refuses to give you enough money for household expenses, even when he has money for other things?
	2. Takes money that you have earned away from you?
	3. Makes important financial decisions without consulting you?
Emotional abuse	Has your current partner, or any other partner ever:
	1. Insulted you or made you feel bad about yourself?
	2. Belittled or humiliated you in front of other people?
	3. Done things to scare or intimidate you on purpose (e.g. by the way he looked at you, by yelling and smashing things)?
	4. Verbally threatened to hurt you or someone you care about?

For each type of violence/abuse, if a woman answers yes to one of more of the questions, then she is recorded has having experienced that form of violence/abuse

A woman is recorded as having experienced physical *and/or* sexual violence (primary outcome) if she answers yes to one or more of the six questions relating to physical violence *and/or* one or more of the three questions relating to sexual violence

### Participant timeline

**Fig. 2:**
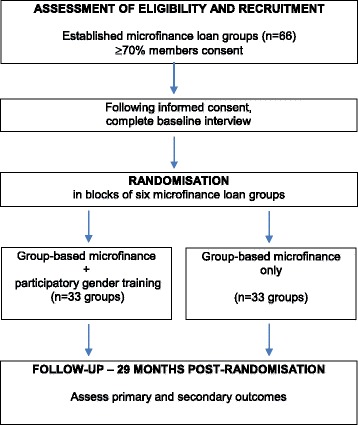
Overview of participant flow

Following enrolment into the study, baseline data are collected from women who have consented to take part. Randomization occurs once all women in a block of six microfinance loan groups have completed the baseline interview. The intervention is delivered over 20 weeks (five months) and women in the both study arms are then followed up 24 months later, i.e. 29 months post-randomization (Fig. 2).

### Sample size

The sample size calculation assumes an estimated prevalence of IPV during the past 12 months of 30% in the comparison arm, based on data from the WHO multi-country study in Tanzania [8]. A sample size of 33 microfinance loan groups per study arm with an average of 20 participants per group (allowing for 10% loss to follow-up) will provide 80% power to detect a reduction of 30% in physical and/or sexual IPV during the past 12 months, and 90% power to detect a reduction of 34%, assuming an intra-cluster correlation of 0.02. Even with an intra-cluster correlation of 0.04, the study will have 80% power to detect a reduction in IPV during the past 12 months of 33%.

### Recruitment of microfinance groups

The study team, in collaboration with BRAC, has identified three neighborhood BRAC branches, out of the seven branches operating across Mwanza city, in which to recruit established microfinance loan groups. Within these three neighborhoods, there are 220 established microfinance loan groups. The study team works closely with BRAC to select groups to approach and invite to take part in the study. Selection of groups to approach is based on factors such as the length of time the group has been established (at least one year), the size of the group (between 15 and 30 active members), and good attendance at the weekly loan meetings, with a good record of loan repayments.

### Allocation method and blinding

Randomization occurs in blocks of six microfinance loan groups. To ensure transparency of the process to the communities, randomization and allocation is a participatory process involving the study team and a representative from each of the six microfinance loan groups to be randomized. Groups are allocated to either intervention or control by tossing a coin. First, representatives from each of the six microfinance groups are randomly divided into two sets (A and B) of three groups. This is done by each representative drawing a folded sheet of paper (with A or B written on it) from a box. One of the representative is asked to call, heads or tails, for her set of three groups to be allocated to the intervention. A study team member then tosses the coin. Given the nature of the intervention, it is not possible to blind participants, or the study team involved in day-to-day operations and delivery of the MAISHA curriculum, after assignment of the intervention. Data analysts will be blinded to allocation.

### Data collection methods – quantitative

The MAISHA study schedule is outlined in Table 3 (adapted from the SPIRIT template [14]). Data are collected at the following time points:

**Table 3:** MAISHA study schedule (based on SPIRIT template [14])

	STUDY PERIOD							
	Enrolment	Allocation	Intervention					Closeout
Time point (months)	*- M1*	*0*	*M1*	*M2*	*M3*	*M4*	*M5*	*M29*
Enrolment								
Eligibility screen	x							
Informed consent	x							
Allocation		x						
Intervention								
No intervention (control)			x	x	x	x	x	
PGT (intervention)			x	x	x	x	x	
Assessments								
*Baseline:*								
Socio-demographics	x							
Physical IPV ^a^	x							
Sexual IPV ^a^	x							
Emotional/psychological IPV	x							
Attitudes about IPV	x							
Disclosure of IPV to others ^a^	x							
In-depth interview ^b^		x						
Focus group discussion ^b^		x						
*Post-intervention:* ^c^								
In-depth interview ^b^							x	
Focus group discussion ^b^							x	
*Follow-up:*								
Socio-demographics								x
Physical IPV ^a^								x
Sexual IPV ^a^								x
Emotional abuse ^a^								x
Attitudes about IPV								x
Disclosure of IPV to others ^a^								x
In-depth interview ^b^								x
Focus group discussion ^b^								x

PGT-participatory gender training; IPV-intimate partner violence;

^a^ Reported experience during past 12 months

^b^ Participants are a purposive sample of women from control and intervention arms – the same women will participate at three time-points

^c^ immediately following completion of the MAISHA curriculum

1. Baseline (prior to randomization) – following informed consent procedures, a face-to-face interview is conducted using a structured questionnaire adapted from the WHO Violence Against Women instrument [8]. The MAISHA questionnaire has seven sections which ask the woman about her: household; partner; income; health; attitudes and social norms; relationship (including experiences of violence); childhood; and about her community. The questionnaire has been translated into Swahili (the national language) and interviews are conducted in private by female interviewers trained in interviewing techniques, gender issues, violence and ethical issues related to research on IPV [15].

2. Intervention – during the 20-week intervention period, the following data are collected: attendance, or not, at the MAISHA curriculum sessions – to understand the “dose” of intervention received; and reasons for non-attendance at the MAISHA curriculum sessions – to understand the potential barriers to attendance.

3. 29 months post-randomization – a face-to-face interview is conducted using a structured questionnaire similar to that used at baseline and following the same procedures.

### Data collection methods – qualitative

A total of 54 in-depth interviews (IDIs) are being conducted with participants. Eighteen women are being purposefully selected from the two study arms to represent women who do and do not report IPV at baseline. A separate team of trained interviewers conduct the IDIs and are blinded as to whether, or not, a woman has reported IPV. Each woman is invited to attend three IDIs – pre-intervention, immediately post-intervention and 24 months post-intervention. The IDIs explore the participants’ life stories and experiences of microfinance, the socio-cultural and structural factors associated with IPV and personal experiences of IPV and its impact on both themselves and their children. For women in the intervention arm, the post-intervention IDIs also explore their views and experiences of the MAISHA curriculum and its impact on their experiences of IPV. Five trial participants from the intervention arm who drop out of the MAISHA curriculum after attending two sessions will be invited to participate in an IDI to explore their reasons for withdrawal from the MAISHA curriculum.

Up to 10 key informant interviews are being conducted with local government and non-government organization officials, police, influential community leaders (e.g. religious leaders) and health care professionals. Interviews are conducted pre-intervention and 24 months post-intervention and explore the wider social and political context for IPV.

Twenty-seven focus group discussions (FGDs) are being conducted – comprising nine FGDs at three time points (pre-intervention, immediately post-intervention and 24 months post-intervention). Six FGDs are being conducted with women in the intervention arm and three with women in the control arm. Where possible the same women (approximately 10 per focus group) are asked to attend at all three time points. The FGDs explore experiences of microfinance and the socio-cultural and structural factors associated with IPV. The post-intervention FGDs with women in the intervention arm also explore their views and experiences of the MAISHA curriculum and its impact on their views of IPV.

FGDs are being conducted with the MAISHA curriculum facilitators to explore their views on the curriculum as a whole and on specific modules, the challenges they have experienced when delivering the sessions, and their perspectives on the impact of the MAISHA curriculum.

The photo voices method is being used to enhance understanding of IPV and intimate relationships. A total of nine women (six from the intervention arm and three from the control arm) are invited to take part immediately post-intervention. Participants receive two days training on using a camera and the ethics of taking photographs in the community before being asked to spend one week photographing everyday lives in their community with a focus on healthy relationships. The participants are then interviewed and asked to provide oral narratives of the photographs they have taken.

Participatory observations are being conducted at selected microfinance loan group meetings and at the MAISHA curriculum sessions, ensuring that each session is observed at least once. Social scientists conduct informal  conversations with study participants to assess their impressions of the curriculum sessions and its immediate impact.

### Data management

Questionnaire data collected from study participants at baseline and at 29 months post-randomization are recorded directly onto a tablet computer. The questionnaire forms have in-built checks to minimize the level of missing data and to minimize entry of erroneous data. The data recorded on the tablet computer are uploaded to the study database daily and checked for missing and/or erroneous data. Any data queries are sent to the team leader to be resolved with the research assistants conducting the interviews.

Attendance at the MAISHA curriculum sessions and reasons for non-attendance are recorded on paper and entered into the study database following double-entry data procedures. Data are checked for missing and/or erroneous data. Any data queries are sent to the team leader to be resolved with the MAISHA curriculum facilitators.

All IDIs and FGDs are recorded with the participants' consent. Hand written notes are taken during the participatory observations of the MAISHA curriculum sessions and microfinance loan group meetings. Audio recordings and hand written notes are transcribed and translated from Swahili (the national language) into English. A sample of the transcripts are checked for quality of transcription and translation. Transcripts are imported to the qualitative analysis package NVIVO (QSR International Pty Ltd, Doncaster, Australia). All visual material, including photographs from the photo voices activities, are imported into the same package.

All study data are stored in secure databases with restricted access. Each participant is allocated a unique study identifier. Names and other identifiers are not recorded in the study database. Paper records – e.g. consent forms, tracking forms with names and contact details – are stored securely in locked filing cabinets in secure offices within the study coordinating center at MITU, which has 24-h security and restricted access.

### Statistical methods

A detailed statistical analysis plan will be prepared prior to follow-up interviews. Data from the baseline interviews will be used to verify the sample size calculations and to identify differences between clusters. The coefficient of variation across clusters will be calculated based on the reported prevalence of IPV. Data from the baseline quantitative interviews will also be used to identify important predictors for IPV and important health-related outcomes, such as poor mental health.

The primary study analysis will adopt an intention to treat approach, assessing the impact of the intervention on women in the intervention arm at 29 months post-randomization (24 months post-intervention), irrespective of whether or not they received the full “dose” (i.e. 10 sessions) of the MAISHA curriculum. Secondary analyses will be conducted to investigate differences in impact according to the dose of the intervention received.

The primary outcome variable (reported experience of a composite of physical and/or sexual IPV during the past 12 months) will be analyzed in a random intercepts logistic regression model to account for the clustered study design, and adjusted for differences in baseline characteristics where relevant. The analysis will be repeated to examine the secondary outcome variables – reported experience of physical IPV, sexual IPV and emotional/psychological abuse during the past 12 months, attitudes towards the acceptability of IPV and, disclosure of violence to others among women who report having experienced physical and/or sexual IPV during the past 12 months. Multiple imputation will be used to simulate missing outcome data. The imputation model will be informed by empirical patterns in the IPV data at baseline and at follow-up. A sensitivity analysis will be conducted, excluding women who participated in the qualitative sub-study (including IDIs, FGDs and photo voices) on the basis that the additional contact of this sub-sample with the study team, as part of these activities, may impact on the effect of the intervention. The analysis will assess if there is any change in the magnitude of the effect.

Steps have been taken to minimize contamination of the control arm, which includes recording women’s attendance at the MAISHA curriculum sessions. The potential for direct and indirect contamination of control arm women will be investigated by asking women during follow-up if they attended any of the MAISHA curriculum sessions or if they have discussed any of the sessions with other women participating in the MAISHA study.

### Safety monitoring

Given that no outcome data (i.e. experiences of IPV) are collected during the five-month intervention period or during the period up to 24 months post-intervention, a data monitoring committee has not been established as no interim analyses are planned. The study is being conducted following the WHO’s guidelines on researching violence against women [15]. Female interviewers for the quantitative baseline and follow-up interviews and for the qualitative IDIs have received training in interviewing techniques, gender issues, violence and ethical issues related to research on IPV. It is anticipated that any harm to women as a result of taking part in the study will be minimal. All participants are provided with information about organizations offering support to women (and their children, if appropriate) experiencing violence and other forms of abuse. Participants who report violence and other forms of abuse are offered counseling by a trained member of the study team and referral to an appropriate organization for ongoing support.

### Auditing

Regular audits of the conduct of the study are carried out by members of the study team. These include checks that participant informed consent procedures have been followed correctly, observation and assessments of facilitation of the MAISHA curriculum sessions, and monitoring of participant attendance at MAISHA curriculum sessions and follow-up of non-attenders.

### Informed consent

Once a microfinance loan group is identified as meeting eligibility, the study team attends the weekly meetings to present information about the study and provides a copy of the participant information sheet (see: Additional file 1) to each of the microfinance loan group members. Each microfinance loan group member meets with a member of the study team to go through the participant information sheet in detail and to allow the microfinance loan group member to ask questions about the study. If the woman agrees to participate and has demonstrated that she understands the study procedures, she is invited to sign the consent form (see: Additional file 1). Participants and key informants who are invited to take part in IDIs are given a participant information sheet providing information about the IDI (see: Additional file 2). A member of the study team meets with the participant/key informant to go through the participant information sheet in detail and to allow the participant to ask any questions. If the participant/key informant agrees to participate in the IDI she/he is invited to sign a consent form (see: Additional file 2). Participants who are invited to take part in an FGD are given an information sheet about the FGD (see: Additional file 3) following the same procedures described above for obtaining informed consent.

### Confidentiality

Participants’ names and any information that could identify them is kept confidential. Women are allocated a unique study identifier. The questionnaires for the quantitative baseline and follow-up interviews are anonymous and responses to questions are entered directly onto a tablet computer. On the same day as the interview, data are uploaded to the secure study database and removed from the tablet computer before the next interview is conducted. Qualitative IDIs are audio recorded with the participants' consent. The recordings are labelled with the study identifier only and are destroyed once the recording has been transcribed and translated to English. All personal identifiers will be destroyed at the end of the study.

### Ancillary and post-trial care

During the 24-month follow-up period following delivery of the intervention, the study team maintains regular contact with participants in order to minimize losses to follow-up. Women who report violence and other forms of abuse during this time are offered support and referred to appropriate organizations for ongoing support post-study.

### Protocol amendments

Since the start of recruitment, there has been one amendment to the protocol approved by the ethics committees. The follow-up period has been extended from 12 months post-intervention to 24 months post-intervention following confirmation of the additional funding required. The study investigators felt that this would be a more appropriate time point at which to assess the effectiveness of the MAISHA curriculum in reducing women’s experience of IPV, and to ensure greater comparability with the IMAGE study. In addition, the secondary outcomes were reviewed and amended to ensure that they were clearly defined, specific and measurable.

### Dissemination policy

The study findings will be widely disseminated through both formal and informal mechanisms. Meetings will be held with participants to inform them of the results of the study. For women in the control arm, information will be provided as to how the MAISHA curriculum will be expanded into their communities, if it is shown to impact on levels of IPV. The study findings will be presented to key stakeholders at local, regional and national level in Tanzania and at relevant regional, national and international conferences and meetings. Reports of the study will be prepared by the study team for submission to peer-review scientific journals. Other strategies to facilitate dissemination of the results of the study will be developed through collaboration with organisations, consortia and forums such as the STRIVE Research Programme Consortium (Tackling the structural drivers of HIV) and the Sexual Violence Research Initiative (SVRI).

## Discussion

The cluster RCT described in this paper (MAISHA CRT01) forms part of the MAISHA study, a programme of research that also includes: a second complementary cluster RCT (MAISHA CRT02) to evaluate the impact of the MAISHA curriculum delivered to newly-formed groups of women who are not engaged in formal group-based microfinance activties; an economic evaluation to evaluate the total costs of the development and implementation of MAISHA CRT01 and MAISHA CRT02; and a cross-sectional survey of the male partners of women taking part in CRT01 to identify risk factors in men associated with IPV (e.g. alcohol use, employment, and abuse during childhood) and to explore whether the intervention delivered to women has impacted on their male partners’ attitudes and behavior.

### Strengths and limitations

A major strength of MAISHA CRT01 is its mixed methods design, utilizing both qualitative and quantitative approaches, to better understand the effects of the intervention and how it is experienced by the participants [16]. Utilizing a randomized design will ensure scientific rigor in the quantitative evaluation of the intervention. Another strength of the study is the large sample size (66 established formal microfinance loan groups), which represents around one third of the established formal microfinance loan groups within the defined study area. Although it is possible that these groups may not be a representative sample of all established formal microfinance loan groups in Mwanza city, the study does not have the resources to collect data on the characteristics of women in non-participating groups to assess the extent of any selection bias. However, it is important to note that any such bias would affect how generalizable the results of the study are to the wider population of women engaged in formal group-based microfinance activities, rather than compromise the internal validity of the study itself. Another limitation, common to studies of complex interventions, is that it will be difficult to unpack which elements of the intervention may or may not have an impact on IPV. An integral part of the MAISHA intervention is that it enables women in microfinance loan groups to have more time together and thereby more time for interaction. Women in the intervention groups meet for longer (either before or after their loan group meeting) on alternate weeks, over a 20-week period, in order to complete the MAISHA curriculum. Whereas, women in the control groups continue with their usual weekly loan group meetings with no additional time for interaction. If an impact on IPV rates is observed in the intervention groups, it may be difficult to determine whether it has resulted from the additional group time or the curriculum, or a combination of both. Data from the complementary qualitative study will be invaluable in exploring women’s experiences of the MAISHA curriculum and format, potential reasons for its success or failure to prevent IPV and variations in impact across groups and/or individual participants.

### Progress and timelines

For MAISHA CRT01, recruitment of 66 established formal microfinance loan groups is complete. Of these, 33 groups were randomly allocated to the intervention arm and 33 groups to the control arm. Delivery of the MAISHA curriculum to the 33 groups allocated to the intervention arm is complete. Baseline interviews with participants indicate a prevalence of physical and/or sexual IPV during the past 12 months of 27% (95% confidence interval: 24% to 29%) [17], confirming the assumption made for the sample size calculation of 30% prevalence of physical and/or sexual IPV during the past 12 months. Follow-up of participants for assessment of the primary and secondary outcomes at 24 months post-intervention is almost complete. Data analysis will be conducted from 2018 onwards and the results of the trial disseminated as described above.

For MAISHA CRT02, formation and recruitment of 66 groups of women not engaged in formal group-based microfinance activities is complete. Delivery of the MAISHA curriculum to the 33 groups allocated to the intervention arm is also complete. Follow-up of women for the primary and secondary outcomes will commence in 2018. A separate paper describing the protocol for MAISHA CRT02 has been prepared.

### Secondary analyses of the MAISHA study datasets

The different components of the MAISHA study will generate a large volume of quantitative and qualitative data on the prevalence of IPV in Mwanza (Tanzania’s second city), risk factors for IPV, the impact of interventions to prevent women’s experience of IPV, attitudes towards the acceptability of IPV and the socio-cultural and structural factors associated with IPV. Secondary analyses of these data are planned, which will include analyses to explore and better understand how the MAISHA curriculum may or may not impact on: the different forms of IPV; patterns of IPV; patterns of communication between couples; and women’s physical and mental health, including sexual behavior. In addition, using data collected from the male partners of CRT01 participants, analyses will explore men’s knowledge and attitudes towards IPV and how these compare with those of women, and whether the MAISHA curriculum delivered to women has any impact on their male partners.

Based on data collected at baseline and at follow-up, analyses will be conducted to explore changes over time: in patterns of IPV experienced by women; in women’s attitudes towards the acceptability of IPV; and, changes in women’s physical and mental health. Structural equation modelling techniques and factor analysis, where relevant, will be used to investigate the hypothesized pathways of IPV, and to verify the pre-conceived theory of change model. Analyses of the qualitative data will include exploration of: the social and political context for IPV; socio-cultural and structural factors associated with IPV; and women’s experiences and views on interventions to prevent IPV.

## Conclusion

In summary, the MAISHA study aims to address the urgent need for rigorous evidence on violence prevention interventions, the need for more data on the different forms of violence, the need to better understand the consequences of violence, such as the impact on the health of women and their families, and the need to better understand the drivers of violence perpetration.

## Additional files

Additional file 1: Participant Information and Consent Form for MAISHA CRT01. Information provided to potential participants, as part of the informed consent process for the MAISHA study, and the informed consent form signed by participants who agree to take part in the study. (DOCX 35 kb)

Additional file 2: Participant Information and Consent Form for MAISHA CRT01 – In-depth Interview. Information provided to potential participants, as part of the informed consent process for participant and key informant in-depth interviews, and the informed consent form signed by participants and key informants who agree to take part in the in-depth interviews. (DOC 49 kb)

Additional file 3: Participant Information and Consent Form for MAISHA CRT01 – Focus Group Discussion. Information provided to potential participants, as part of the informed consent process for focus group discussion, and the informed consent form signed by participants who agree to take part in the focus group discussions. (DOC 51 kb)

## Abbreviations

BRAC: Bangladesh Rural Advancement Committee
DFID: Department for International Development
FGD: focus group discussion
IDI: in-depth interview
IMAGE: Intervention with Microfinance for AIDS & Gender Equity
IPV: intimate partner violence
LSHTM: London School of Hygiene & Tropical Medicine
MITU: Mwanza Intervention Trials Unit
NIMR: National Institute for Medical Research
RCT: randomized controlled trial
SPIRIT: Standard Protocol Items: Recommended for Intervention Trials
SVRI: Sexual Violence Research Initiative
WHO: World Health Organization
