# Supplementary material for: A cluster randomized controlled trial to assess the impact on intimate partner violence of a 10-session participatory gender training curriculum delivered to women taking part in a group-based microfinance loan scheme in Tanzania (MAISHA CRT01): study protocol
Source: BMC Womens Health. 2018 Apr 2;18:55. doi: 10.1186/s12905-018-0546-8 (PMC5879641; doi:10.1186/s12905-018-0546-8)
Supplement: Supplementary file 1 — Participant Information and Consent Form for MAISHA CRT01. Information provided to potential participants, as part of the informed consent process for the MAISHA study, and the informed consent form signed by participants who agree to take part in the study. (DOCX 35 kb) [file 12905_2018_546_MOESM1_ESM.docx]

**A cluster randomised controlled trial to assess the incremental impact on intimate partner violence of adding a 10-session participatory gender training programme to an existing microfinance intervention for women in Tanzania (MAISHA CRT01): study protocol**

**PARTICIPANT INFORMATION AND CONSENT FORM FOR MAISHA CRT01**

Version 1.2, 23 Sep 2014

**Introduction**

We are conducting research on an important issue related to health and healthy relationships. We would like to find out if you are interested to participate in this project. This research is being conducted by the National Institute for Medical Research (NIMR), Mwanza centre, and the Mwanza Intervention Trials Unit (MITU) in collaboration with the London School of Hygiene & Tropical Medicine (LSHTM). We have been approved to conduct this research by the Tanzania Ministry of Health and Social Welfare and by the London School of Hygiene & Tropical Medical ethics committee.

This form provides information about the study procedures. After reading and talking about the information provided with the study staff, you will be able to decide whether you want to take part in this study. If you decide to take part, we will ask you to sign this consent form.

Please note that:

- Your decision to take part is entirely voluntary. It is completely up to you to decide whether to take part in this project.
- You may decide not to take part, and not lose any of your rights or benefits such as the standard medical care you usually receive.
- If you decide to take part, you may drop out at any time, for any reason, without losing any rights or benefits.

**What is the purpose of the study?**

The purpose of this study is to explore ways to improve relationships and health more generally. In this study, a number of women groups receiving small financial loans, using a local organization called BRAC, will be selected and invited to take part in the study. Once the groups join the study, they will be divided without following any specific order (i.e. by chance), to either receive training on gender issues using methods which allow active participation during the training sessions, or not receiving such training.

After two years of implementing these activities, we will assess the impact on relationships for the women in the study as well as other health related outcomes. We will also measure changes on the ability of women to have control over their everyday lives, and the economic and health benefits. We will also assess the cost of implementing these activities against their effectiveness.

**RESEARCH PROCEDURES**

If you decide to participate, you will be asked to take part in the following study procedures. You are free to withdraw from any of these procedures at any time:

1. Preparation group meetings

As part of the study, you have been asked to attend four group meetings with up to 30 other women. At the first meeting, you will be provided with an explanation of the study, and you will receive this form and research staff will go through it with you and explain the study procedures in detail. During the meeting, you will have time to ask any questions you might have from reading this form. Every group that takes part in this study has the chance to be allocated to take part in the activities discussed above.

Please note all those who agree to participate in this study will be interviewed at the beginning and towards the end of the study. Participation by your group in any of the activities will be determined by chance. This means that your group will have equal chance of taking part in any of the activities described under (2) below.

2. Group activities

The group activities you could be participating in are:

- If your group is selected to receive training on gender, your group will be provided with 10 training sessions covering important issues such as the role of women in your community, (domestic) violence, HIV and other health related issues at a place and time convenient to all the group members. This could be after your weekly BRAC loan group meetings or at a different time. The sessions will be conducted over a period of up to 6 months. These sessions will be led by an experienced facilitator and each session will last about two hours. All women in these groups will be asked to attend an interview before and after the training sessions.

OR

- If your group is not selected to receive training on gender, you will only be asked to attend two interviews at the beginning of the study and towards the end (first interview and second interview).

3. First Interview

After you have agreed to take part in the study, a researcher will visit you at an agreed location and conduct an interview. You will be asked a series of questions about yourself, your health and your personal experiences in relationships.

This interview will be held by a trained interviewer and at a place convenient to you. Everything you tell us will be kept confidential. At any time during the interview you can refuse to answer questions or withdraw from the interview. As the interview will take between two and three hours to complete, you may also ask for a break if you are tired or something requires your immediate attention. This interview is very important for this study and if you agree to participate in this study you also agree to be interviewed.

4. Second interview

After your group has completed the assigned activity, you will be contacted a year later to be interviewed for a second time. Similar to the first interview, you will be asked a series of questions about the same topics. The purpose of this interview is to see if attending the group activities has resulted in any changes in your life. As for the first interview, this interview will be conducted at a place convenient to you and all your answers will be kept confidential and you may refuse to answer if you are not comfortable with some questions. This second interview will also take about two to three hours to complete. Again, this interview is very important and if you agree to take part in this study you also agree to this second interview.

5. Other interviews

You may be asked at any time during the study to attend an individual or group interview or other activities such as mapping your community or making a photographic record of your community in order to better understand your knowledge and perceptions of healthy relationships and what you think about the activities you are taking part in. If you are invited to attend other interviews you will be provided with a full explanation and you will sign a separate consent form. It is important to know that your participation in this study does not oblige you to take part in these other activities and interviews.

**Results of the study**

After we have completed the study and analyzed the information, we will inform you of the outcomes of the study through meeting(s) to be organized in Mwanza city. We will also inform local and national government of the findings. Depending on the outcome of the study BRAC may decide to continue to provide gender training session for other women in Mwanza.

**How will the information I give be kept private?**

All information collected in this study will be kept securely and confidential in a locked location. Your name and details will not be recorded on the notes written during the discussions. Analysis will only be done using the study number and your identity will remain private and confidential, unless we are required by law to release the information. Reports about the study and results will be presented to the organizations that are working together for this study and at scientific meetings. Results of this study may also be published in scientific journals. All presentations and publications of the findings of this study will not include any information which allows you to be identified as one of the study participants.

**What are the risks, stress or discomfort of taking part in this study?**

We do not expect that you will experience any harm by taking part in this study. However, some of the questions may be sensitive and you may feel embarrassed to discuss them with other people in the group. You can stop the interview or withdraw from the study if you feel uncomfortable.

If you decide you do not want to take part this will not affect this community being offered small financial loans or training on gender issues in the future. If you have any problems as a result of being in the study then you should discuss this with one of the persons named below.

**What are the benefits of participating?**

For those who are attending the training may benefit from the information about how to improve your relationship and your health more generally. For those who only attend the interview this study may have no direct benefit to you, however this research will help us to prepare training activities and materials that are acceptable and that may bring positive changes to the healthiness of relationships in this community. Therefore, your participation in this study will provide information which could help you and others in your community in the future. If you are subject to any violence during the course of the study, the study staff will provide information about available services within your community and refer you to the services if appropriate.

**Are there costs associated with participating in this study?**

There are no costs to you for participating in this study. For those of you who are in the group that will attend training, you will be given a reimbursement of Tanzanian shillings 2,500 after each training session. Everyone else who participates in the study will receive Tanzanian shillings 5,000 after each interview. This is compensation for your time, inconvenience and other costs which may be related to your participation in the study.

**Length of participation**

You will be asked to be interviewed around a week after signing this consent from. If you are selected to attend training this start around one month after you sign this consent from and will take place up to 6 months. The second interview will be conducted at some time later in 2015. It is therefore likely that your study participation will be approximately 24 months.

**Whom can I contact if I have questions or need additional information?**

We would like to answer all your questions. If you have any questions now, please ask us. If you have any questions later, you can also contact Dr Saidi Kapiga (Co-Investigator of this study and the Scientific Director of the Mwanza Intervention Trials Unit – MITU) or Dr Gerry Mshana (Co-Investigator of this study) at the following address:

Mwanza Intervention Trials Unit

National Institute for Medical Research

P.O. Box 11936

Mwanza, Tanzania

Telephone: 028-250 0019

If at any time you have any questions regarding your rights as a participant in this research study, you may contact Ms Joyce Ikingura at the address shown below:

Medical Research Coordinating Committee

National Institute for Medical research

P.O. Box 6953

Dar es Salaam, Tanzania

Telephone: 022-212 1400

**Agreement to join the study**

I have read this form, or had it read and explained to me. I understand the information and was able to ask all my questions. I have been given a copy of this form.

I voluntarily agree to participate in this study by signing below. If I am illiterate, I agree that a witness will underwrite and sign on my behalf.

Participant is illiterate Participant is literate

in Swahili in Swahili

Participant name (**print**) Participant signature/Thumbprint Date

Name of study staff conducting Study Staff signature Date

consent discussion (**print**)

Witness name* **(print)** Witness signature* Date*

(***Needed only if participant is illiterate**)
