# Supplementary material for: A cluster randomized controlled trial to assess the impact on intimate partner violence of a 10-session participatory gender training curriculum delivered to women taking part in a group-based microfinance loan scheme in Tanzania (MAISHA CRT01): study protocol
Source: BMC Womens Health. 2018 Apr 2;18:55. doi: 10.1186/s12905-018-0546-8 (PMC5879641; doi:10.1186/s12905-018-0546-8)
Supplement: Supplementary file 3 — Participant Information and Consent Form for MAISHA CRT01 – Focus Group Discussion. Information provided to potential participants, as part of the informed consent process for focus group discussion, and the informed consent form signed by participants who agree to take part in the focus group discussions. (DOC 51 kb) [file 12905_2018_546_MOESM3_ESM.doc]

**A cluster randomised controlled trial to assess the incremental impact on intimate partner violence of adding a 10-session participatory gender training programme to an existing microfinance intervention for women in Tanzania (MAISHA CRT01): study protocol**

**PARTICIPANT INFORMATION AND CONSENT FORM FOR MAISHA CRT01**

**FOCUS GROUP DISCUSSION**

Version 1.1, 24 March 2015

**INTRODUCTION**

We are conducting research on an important issue related to health and healthy relationships. This research is being conducted by the National Institute for Medical Research (NIMR), Mwanza centre and the Mwanza Intervention Trials Unit (MITU) in collaboration with the London School of Hygiene & Tropical Medicine (LSHTM). We have been approved to conduct this research by the Tanzania Ministry of Health and Social Welfare and by the LSHTM ethics committee.

The purpose of the study is to explore ways to improve relationships and health more generally. In this study a number of BRAC groups will be selected by chance to receive training on gender issues using methods which allow active participation during the training sessions. Other women will be selected to form a group and by chance to receive training on gender issues also using methods that allow active participation during the training sessions. For these women, separate training sessions on issues related to gender issues will be organized for their male partners if these women agree to their partners being involved. In order to measure whether loans and training or training alone impact on improving healthy relationships and health; we will also recruit a number of women to form a group who will only be asked to attend discussions. These women will not receive either small financial loans or training on gender issues, during this study.

After one year of implementing these activities, we will assess the impact on relationships for the women in the study as well as other health related outcomes. We will also measure changes on the ability of women to have control over their everyday lives, and the economic and health benefits. We will also assess the cost of implementing these activities against their effectiveness.

**PARTICIPATORY GROUP DISCUSSIONS**

As part of the study we are planning to conduct participatory group discussions to help us gain more understanding of issues related to healthy relationships. This will involve detailed discussions and involvement with activities about health and healthy relationships in a group of individuals.

We would like to hear about your views and experiences about health and healthy relationships. We would also like your views on other issues including the roles of women and men in financial issues, family life, as well as your views on violence against women. If you are part of the main study and have attended gender training or received microfinance we would also like to hear your views and experiences of these.

Information from these participatory group discussions will help us to gain an in-depth understanding of health and healthy relationships and evaluate the effect of the project activities on improving these. If you are part of the main study and have attended gender training or received microfinance we would also like to understand the impact of these on your health and relationships.

After you have had all your questions answered and feel you understood what you will have to do, you will be asked to sign, or put your thumb print on this consent form. The researcher will ask you for permission to record the discussion and activities. If you do not want to be recorded but others in the group agree, you will be free to decline participating in the discussion. The discussions will be informal and you will be encouraged to talk freely about anything that you feel is related to the questions about health and healthy relationships.There are no right and wrong answers and all your views will be respected.

The discussion should take no longer than 2 hours and will be conducted in a private place suitable to you. If the discussion takes longer and you need to leave to attend other duties, please feel free to notify the research moderator or the note taker.

**DO I HAVE TO TAKE PART?**

This form provides information about the procedures. After reading and talking about the information provided with the researcher, you will be able to decide whether you want to take part in this study. If you decide to take part, we will ask you to sign this consent form.

Please note that:

- Your decision to take part is entirely voluntary. It is completely up to you to decide whether to take part in this participatory group discussion.
- You may decide not to take part, and not lose any of your rights or benefits.
- If you decide to take part, you may drop out at any time, for any reason, without losing any rights or benefits.

**WHY HAVE I BEEN CHOSEN TO TAKE PART IN THE STUDY?**

You are invited to participate in a participatory group discussion because you are participating in the main study or because you live in the neighbourhood area where the main study is being conducted. We hope that you will be interested to participate and provide information relevant to the research.

**WHAT WILL YOU BE ASKED TO DO IF YOU DECIDE TO TAKE PART IN THE PARTICIPATORY GROUP DISCUSSION?**

If you decide to participate in this participatory group discussion, you may be asked to take part in other discussions in the future. You will be contacted and requested for consent to participate before these participatory group discussions. Your participation in these other discussions will be completely voluntary and you can decide not to participate in the future even if you agreed to participate today. You will be asked to participate in the discussions after signing the consent form. If you are invited to attend further discussions they will be conducted in about six month’s time and a year after this discussion. It is therefore likely that your study participation will be for approximately 18 months.

**How will the information I give be kept private?**

Your contact details will be confidential and only be available to the staff involved in the study. The copy of the recording will not have your names attached to it. All information collected in this study will be kept securely and confidential in a locked location. Your names and details will not be recorded on the notes written during the discussions or in reports from the study and your identity will remain private and confidential. Reports about this study may quote some of the words you tell us. Any words that you have told us during the discussion will not have your name attached to them.

**What are the risks, stress oR discomfort of taking part in this discussion?**

We do not expect that you will experience any harm by taking part in this discussion. However, some of the questions may be sensitive and you may feel embarrassed to discuss them with others in a group. You are free not to answer any questions or to withdraw from this discussion if you feel uncomfortable.

If you decide you do not want to take part this will not affect this community being offered small financial loans or training on gender issues in the future. If you have any problems as a result of being in the study then you should discuss this with one of the persons named below.

**WHAT ARE THE BENEFITS OF PARTICIPATING?**

We hope that the information gained from the discussions will improve our understanding of ways to improve healthy relationships in the community and reduce violence against women.

**Are there costs associated with participating in this study?**

There are no costs to you for participating in this study. However, everyone who participates in this study will receive Tanzanian Shillings 5,000 as a contribution towards the cost of your travel and time.

**HOW WILL I HEAR THE RESULTS OF THE STUDY?**

After we have completed the research we will inform you of the outcomes of the main study through meetings which will be organized in Mwanza city. We will also inform local and national government of the findings.

**Whom can I contact if I have questions or need additional information?**

We would like to answer all your questions. If you have any questions now, please ask us. If you have any questions later, you can also contact Dr Saidi Kapiga (Co-Investigator of this study and the Scientific Director of the Mwanza Intervention Trials Unit (MITU) or Dr Gerry Mshana (Co-Investigator of this study) at the address:

Mwanza Intervention Trials Unit

National Institute for Medical Research

P.O. Box 11936

Mwanza, Tanzania

Telephone: 028-250 0019

If at any time you have any questions regarding your rights as a participant in this research study, you may contact Ms Joyce Ikingura at the address shown below:

Medical Research Coordinating Committee

National Institute for Medical research

P.O. Box 6953

Dar es Salaam, Tanzania

Telephone: 022-212 1400

### WHAT AM I REQUIRED TO DO?

If you agree to participate in this research, please sign below.

**Agreement to join the study**

I have read this form, or had it read and explained to me. I understand the information and was able to ask all my questions. I have been given a copy of this form.

I voluntarily agree to participate in this study by signing below. If I am illiterate, I agree that a witness will underwrite and sign on my behalf.

Participant is illiterate Participant is literate

in Swahili in Swahili

Participant name (**print**) Participant signature/Thumbprint Date

Name of study staff conducting Study Staff signature Date

consent discussion (**print**)

Witness name* **(print)** Witness signature* Date

(***Needed only if participant is illiterate**
